# Black phosphorus: a two-dimension saturable absorption material for mid-infrared Q-switched and mode-locked fiber lasers

**DOI:** 10.1038/srep30361

**Published:** 2016-07-26

**Authors:** Jianfeng Li, Hongyu Luo, Bo Zhai, Rongguo Lu, Zhinan Guo, Han Zhang, Yong Liu

**Affiliations:** 1 State Key Laboratory of Electronic Thin Films and Integrated Devices, School of Optoelectronic Information, University of Electronic Science and Technology of China (UESTC), Chengdu 610054, China; 2 SZU-NUS Collaborative Innovation Center for Optoelectronic Science and Technology, Key Laboratory of Optoelectronic Devices and Systems of Ministry of Education and Guangdong Province, College of Optoelectronic Engineering, Shenzhen University, Shenzhen 518060, China

## Abstract

Black phosphorus (BP) as a novel class of two-dimension (2D) materials has recently attracted enormous attention as a result of its unique physical and chemical features. The remarkably strong light-matter interaction and tunable direct band-gap at a wide range make it an ideal candidate especially in the mid-infrared wavelength region as the saturable absorber (SA). In this paper, the simple and effective liquid phase exfoliation (LPE) method was used to fabricate BP. By introducing the same BP SA into two specifically designed rare earth ions doped fluoride fiber lasers at mid-infrared wavebands, Q-switching with the pulse energy of 4.93 μJ and mode-locking with the pulse duration of 8.6 ps were obtained, respectively. The operation wavelength of ~2970 nm for generated pulse is the reported longest wavelength for BP SA based fiber lasers.

In the past decade, rapid progress has been made on the mid-infrared laser sources covering from 2 μm to 20 μm as a result of their widespread and potential applications^1^. More specifically, they are of key significance for the fields such as environmental monitoring and pollution control, detection of water and soil contaminants, food quality control, agriculture and life sciences, invasive disease diagnosis and therapy through breath analysis, etc^2^,^3^,^4^,^5^,^6^,^7^,^8^, since most molecular fundamental vibrational absorptions are located in this wavelength region. Currently, several different approaches e.g., solid-state lasers^9^, quantum cascade lasers (QCL)^10^, difference frequency generation (DFG)^11^, sum frequency generation (SFG)^5^, optical parametric oscillator (OPO)^12^, etc. have been adopted to demonstrate the mid-infrared emissions at different sub-ranges. As another new platform, the mid-infrared laser producing from optical fibers is attracting increasing attention recently because of its outstanding merits such as excellent beam quality, good heat dissipation, high slope efficiency, etc^13^. The continuous improvement of the infrared glass (e.g., fluoride^14^, chalcogenide^15^, fluorotellurite^16^, etc.) fiber drawing technology has also spurred the development of mid-infrared fiber lasers. Until now, their wavelengths have been typically extended to 3 μm and some even to near 4 μm spectral regions stimulated by the potential applications in plastic and polymer processing, laser surgery, laser radar, etc^14^,^15^,^17^,^18^,^19^,^20^,^21^,^22^. Despite the achieved success in power enhancement of continuous wave (CW) state where the highest level at 3 μm waveband has been recorded as 30 W^14^, the relative investigations on pulse generation by either Q-switching or mode-locking, especially implemented in compact and robust passive schemes with the help of saturable absorbers (SAs), is only recent^23^,^24^,^25^,^26^,^27^,^28^,^29^,^30^,^31^,^32^,^33^.

Concerning their matured fabrication technology, semiconductor saturable absorber mirror (SESAM) and Fe^2+^:ZnSe crystal were most early used in pulse generation of fiber lasers in 3 μm spectral region^23^,^24^,^25^,^26^,^27^,^28^,^29^,^30^,^31^. Currently, the reversely designed InAs-based SESAM has been employed to demonstrate the passive Q-switching of 2.97 μm singly Ho^3+^-doped^23^, passive switching of 2.87 μm Ho^3+^/Pr^3+^ co-doped^24^, high power passive mode-locking of 2.8 μm Er^3+^-doped^25^ and even dual wavelength (3 μm and 2.1 μm) passive switching of cascaded Ho^3+^-doped^26^ fluoride fiber lasers. Moreover, InAs material as SA was also used to mode lock the 2.86 μm Ho^3+^/Pr^3+^ co-doped fluoride fiber laser in a ring cavity^28^. However, narrow operation bandwidth mainly limited by the band-gap of InAs material and complex package procedure hindered its applications in longer wavelength region. Meanwhile the bulk Fe^2+^:ZnSe crystal was also used to perform both Q-switching and mode-locking in this wavelength region based on Er^3+^- or Ho^3+^-doped fluoride fiber in different cavity arrangements^29^,^30^,^31^. Although its contribution to high-performance pulse generation is worthy complimenting, the inherent free-space structure limited its future application potential in all-fiber scheme. Most recently, the mode-locking of Er^3+^-doped fluoride fiber laser by the way of traditional nonlinear polarization rotation (NPR) has been also experimentally demonstrated at 2.8 μm. The temporal durations of the yielded pulses were firstly narrowed to the level of several hundreds of femtoseconds at this waveband albeit with comparatively complicated alignment^32^,^33^.

In recent years, the rise of two-dimensional (2D) materials mainly focusing on graphene, topological insulator (TI) (e.g., Bi_2_Te_3_, Bi_2_Se_3_, Sb_2_Te_3_, etc.) and transition metal dichalcogenides (TMDCs) (e.g, MoS_2_, MoSe_2_, WSe_2_, WS_2_, etc.) has provided a great opportunity for new-generation optoelectronic devices as a result of their outstanding physical and chemical properties^34^. Their broadband absorption potential and ultrafast carrier dynamics make them ideal SAs for the formation of infrared pulse. Graphene, as the pioneer of this family, has been widely used in the pulsed fiber lasers in the near-infrared spectral region of 1~2 μm^35^,^36^,^37^,^38^,^39^,^40^,^41^, and also demonstrated its potential in both Q-switching and mode-locking in 3 μm wavelength region recently^42^,^43^. Though it possesses wavelength-independent saturable absorption as a result of the absence of band-gap, the low modulation depth resulted from its low absorption efficiency (2.3% per layer) is not suitable for the pulsed fiber lasers^44^. Lately, TMDCs have been also applied in the pulsed fiber lasers because of their unique absorption^45^,^46^,^47^,^48^,^49^,^50^,^51^, but the relatively large direct band-gaps corresponding to the visible spectrum range limits their applications in infrared region. Though the introduction of some suitable defects could extend their operation wavelengths to some extent, it is still difficult for them to perform the mid-infrared operation thus the current longest effective wavelengths have been only demonstrated around 2 μm^52^,^53^. Besides, the preparation process would become extremely difficult as well. TI as another novel class of Dirac material not only has a nontrivial narrow band-gap but also possesses a gapless metallic state in its edge/surface^54^. The small band-gap combined with its merits of large modulation depth and high damage threshold makes it feasible as an effective SA in mid-infrared spectral region^55^,^56^. Recently, Q-switching with the aid of TI has been successfully performed in fiber lasers around 3 μm^57^. However, it has the drawback of complex preparation process as a result of compound with two different elements.

Black phosphorus (BP), as another novel 2D material, recently has attracted enormous attention owing to its great potential in electronics and optoelectronics applications^58^,^59^,^60^. As the most thermodynamically stable allotrope of phosphorus, BP possesses a high mobility. Similar to the graphene, BP is a layered material with basic cells of honeycomb structure in which the different layers are stacked together with the aid of weak van der Walls forces^61^. Different from the other 2D materials mentioned above, BP has a unique direct band-gap which is layer number dependent^62^. Specifically, its band-gap can be tuned from ~0.3 eV (bulk) to ~2 eV (monolayer) flexibly by changing the layer number hence filling up the gap between the zero band-gap of graphene and large band-gaps of TMDCs. The band-gap-controllable feature is as well helpful for its optoelectronic applications especially in near- and mid-infrared spectral regions. As the motivation that BP only comprises one element component and has a direct band-gap same as graphene, it is natural to consider whether BP can be also used as SA for pulse generation. Recently, a series of experimental demonstrations revealed the saturable absorption of BP in visible and infrared spectral region spanning from 400 nm to 2 μm indicating its broadband potential in pulse generation^63^. Lately, the Q-switched and mode-locked fiber lasers at 1.55 μm using BP as the SA were reported for the first time with quite excellent performance^64^,^65^,^66^. Since then, the relative efforts have been deployed with the operation wavelength extended to 2 μm^67^. Very recently, the Q-switching and mode-locking of 2.8 μm Er^3+^-doped fluoride fiber laser based on BP were also successfully demonstrated with the wavelengths extended towards the mid-infrared region^68^,^69^, but detailed system structure designs were absent.

In this paper, the BP 2D material was fabricated using the cost-effective liquid phase exfoliation (LPE) method, then a series of material characterizations based on Raman spectrum measurement, transmission electron microscope (TEM), atomic force microscope (AFM) were performed while its saturable absorption property was also investigated in 2 μm spectral region. By specifically designing cavity structure of two fluoride fiber lasers, Q-switching and mode-locking were obtained, respectively based on the same BP whose feasible operation wavelength was firstly further extended to near 3 μm region. Besides, laser performances including temporal waveform, output power, pulse energy, pulse duration, repetition rate, RF spectra were also given. The results indicate that BP could be developed as an effective SA used for pulse generation in 3 μm mid-infrared spectral region.

## Results

### Characterizations of black phosphorus

**Figure 1:**
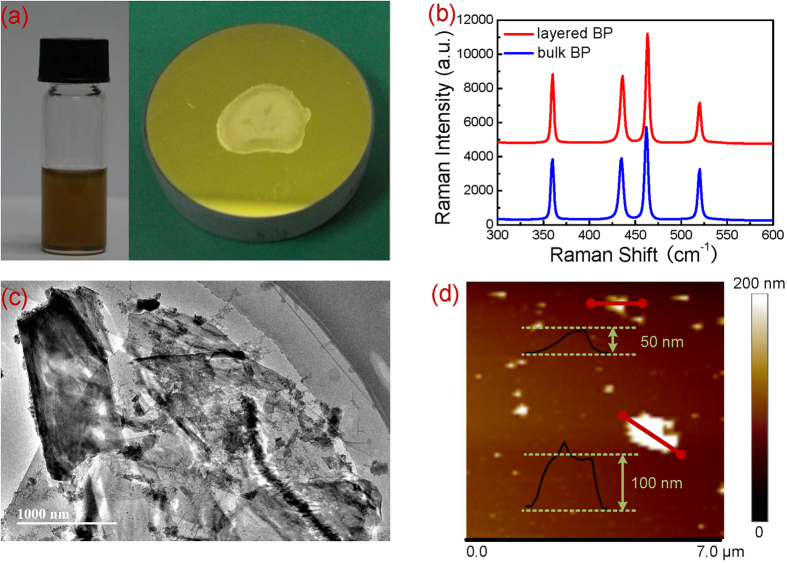
Characterizations of the used BP material. (**a**) Layered BP solution; (**b**) Raman spectra of bulk and layered BPs; (**c**) TEM image of layered BP; (**d**) AFM image of layered BP.

The layered BP was fabricated in a basic-NMP solvent exfoliation method which can be evenly dispersed in NMP, as shown in Fig. 1a. While the fabricated free-space BP SA based on the Au mirror was also shown in Fig. 1a. After sonication in NMP, bulk BP can be exfoliated into layered structure. Firstly, Raman spectrum measurement was taken on the layered BP on silicon wafer. As can be seen from Fig. 1b, except the standard Raman peak of silicon at 520.7 cm^−1^, there are three typical Raman peaks located at 361 cm^−1^, 437 cm^−1^ and 464 cm^−1^ which correspond to BP’s out-of-plane vibration mode A_g_^1^, in-plane vibration modes B_2g_ and A_g_^2^, respectively. It can be also seen that the Raman spectrum of the as-prepared layered BP is almost same as that of bulk BP indicating its relatively large thickness. Moreover, the TEM was employed to characterize the morphology of the layered BP. As can be seen from Fig. 1c, the exfoliated BP is in the form of layered structure. Plenty of BP sheets are stacked together. Besides, the absolute thickness of the layered BP was also characterized by the way of AFM. As is shown in Fig. 1d, the thickness of most of the layered BP is ranging from 50 nm to 100 nm which corresponds to a band-gap of 0.34 eV^70^. It means our as-prepared BP can be operated well in the wavelength region below 3.68 μm.

### Saturable absorption property of black phosphorus

**Figure 2:**
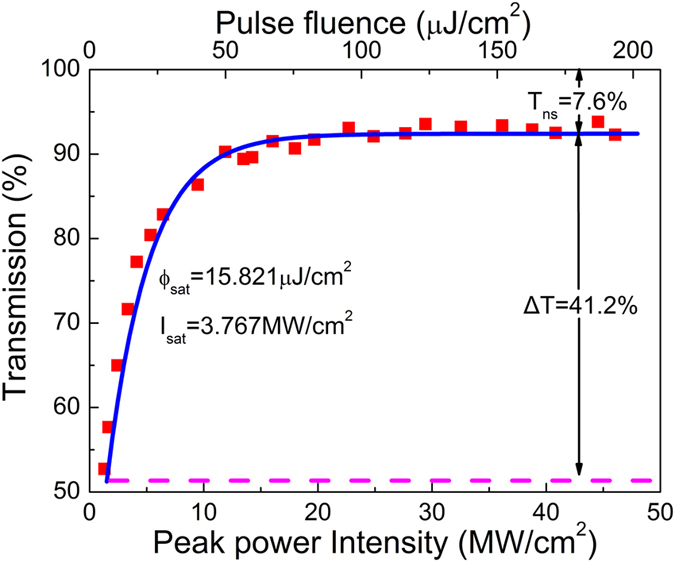
Nonlinear transmission of BP SA as a function of peak power intensity and pulse fluence. The red points are the experimental data and the blue line is the fitting result.

Figure 2 shows the absolute transmissions of the BP SA as the varied incident peak intensity and pulse fluence. It is observed that the transmission increases sharply and then gradually saturates with incident peak intensity or pulse fluence. The phenomenon suggests the saturable absorption of the BP sample. The relative parameters were achieved by fitting the data using the following formula:


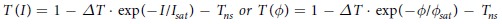


where *T(I)* or *T(ϕ)* is the transmission, *ΔT* is the modulation depth, *I* and *ϕ* are the incident peak intensity and pulse influence, respectively, *I*_*sat*_ and *ϕ*_*sat*_ are the saturation peak intensity and pulse fluence, respectively, and *T*_*ns*_ is the non-saturation loss. The calculated saturation peak intensity of 3.767 MW/cm^2^ matched well with 4.56 MW/cm^2^ measured at 1930 nm^63^. The corresponding saturation pulse fluence of 15.821 μJ/cm^2^ was also reasonably a little higher than 9 μJ/cm^2^ measured at 2.8 μm^69^. The modulation depth and non-saturation loss were 41.2% and 7.6%, respectively.

### Mid-infrared pulsed fiber lasers

**Figure 3:**
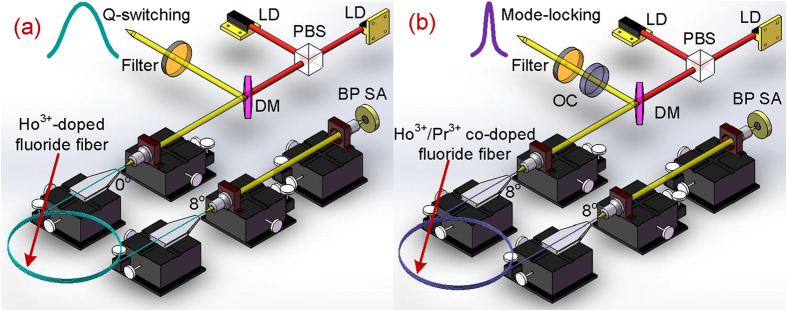
Experimental setup of pulsed fluoride fiber lasers based using BP SA. (**a**) Passively Q-switched Ho^3+^-doped fluoride fiber laser. (**b**) Passively mode-locked Ho^3+^/Pr^3+^ co-doped fluoride fiber laser. LD is the laser diode, PBS is the polarized beam splitter, DM is the dichroic mirror, OC is the output coupler.

**Figure 4:**
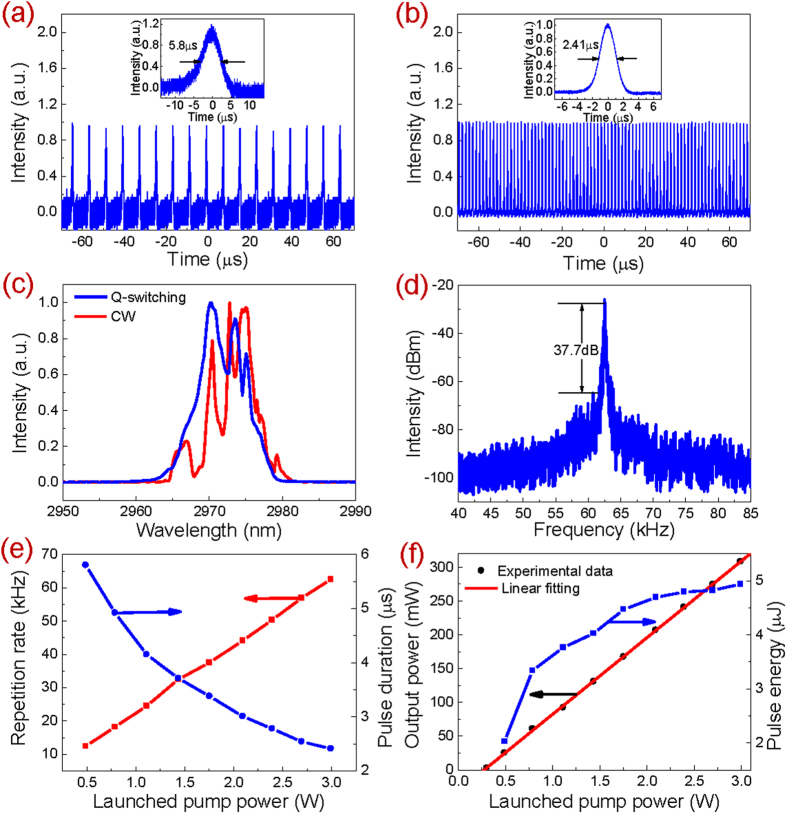
Output performances of passively Q-switched Ho^3+^-doped fluoride fiber laser. (**a**) Pulse train and single pulse waveform (inset) at the launched pump power of 489.3 mW; (**b**) Pulse train and single pulse waveform (inset) at the launched pump power of 2.99 W; (**c**) Optical spectra at the launched pump power of 2.99 W; (**d**) RF spectrum at the launched pump power of 2.99 W. (**d**) Repetition rate and pulse duration as a function of the launched pump power; (**e**) Output power and pulse energy as a function of the launched pump power.

In this section, the output performances of the mid-infrared pulsed fiber lasers as shown in Fig. 3a,b were demonstrated, respectively. In the system as shown in Fig. 3a, CW laser was firstly generated as the launched pump power was increased to 302.6 mW. Once it reached 489.3 mW, stable Q-switching occurred yielding the repetition rate of 12.43 kHz and pulse duration of 5.8 μs as shown in Fig. 4a. The stable Q-switching state can be maintained until the available maximum launched pump power of 2.99 W as shown in Fig. 4b. At this pump level, the pulse duration and repetition rate were measured to be 2.41 μs and 62.5 kHz, respectively. The pulse amplitude fluctuation of ±2% suggested the high stability. Figure 4c shows the measured optical spectrum of the Q-switched pulses. The center wavelength of 2970.3 nm and FWHM of 7.06 nm were achieved. At this moment, if the terminated focused beam spot was moved onto the clean region of the Au mirror where no BP was coated, the laser immediately switched to the CW operation state no matter how we adjusted the Au mirror, thus excluding the possibility of self-pulsing. For comparison, the optical spectrum of the CW laser was also measured as shown in Fig. 4c. It is observed that the center wavelength was red-shifted to 2972.8 nm as a result of the decreased intra-cavity loss induced lower initial Stark manifold of the ^5^I_6_ energy level. Meanwhile, the FWHM was narrowed to 4.5 nm owing to the less required Fourier spectral components for CW emission. The similar phenomenon was observed in our previous experiment where the same fiber was used^57^. Furthermore, the RF spectrum of the Q-switched pulses at the launched pump power of 2.99 W was measured as well at a scanning span of 45 kHz with a resolution bandwidth of 100 Hz, as shown in Fig. 4d. The SNR of 37.7 dB located at the general level of passively Q-switched fiber lasers in this wavelength region^23^,^30^,^31^,^43^,^57^. Figure 4e shows the measured repetition rate and pulse duration as a function of the launched pump power. The repetition rate increases almost linearly from 12.43 kHz to 62.5 kHz with the launched pump power increasing from 489.3 mW to 2.99 W owing to the faster population built-up on ^5^I_6_ level. Meanwhile the pulse duration decreases from 5.8 μs to 2.41 μs non-linearly as a result of more population density accumulation on ^5^I_6_ level. Note that both of them are the typical features of passive Q-switching. Figure 4f shows the measured output power and calculated single pulse energy as the varied launched pump power. It is observed that the output power increases almost linearly while the pulse energy increases nonlinearly as the increased launched pump power. Finally, the maximum output power of 308.7 mW at a slope efficiency of 11.35% and single pulse energy of 4.93 μJ were achieved which were only limited by the available pump power.

**Figure 5:**
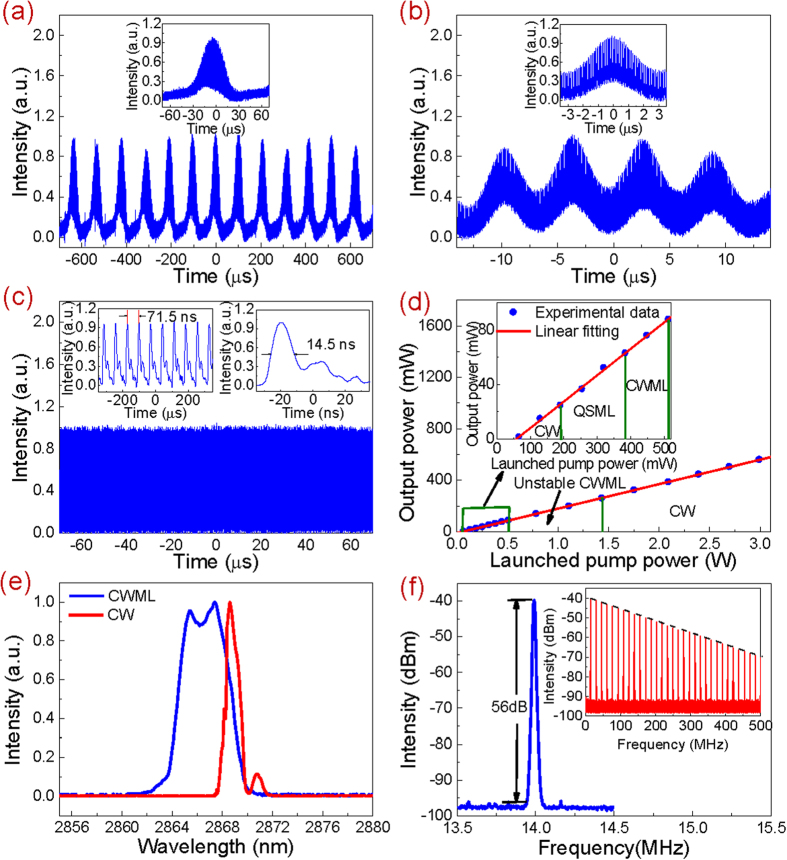
Output performances of passively mode-locked Ho^3+^/Pr^3+^ co-doped fluoride fiber laser. (**a**) Pulse train and single pulse waveform (inset) at the launched pump power of 190.1 mW; (**b**) Pulse train and single pulse waveform (inset) at the launched pump power of 383.42 mW; (**c**) Pulse train and waveforms (inset) once the launched pump power beyond 383.42 mW; (**d**) Output power as a function of the launched pump power; (**e**) Optical spectra at the launched pump power of 512.3 mW; (**f**) RF spectra at the launched pump power of 512.3 mW.

**Figure 6:**
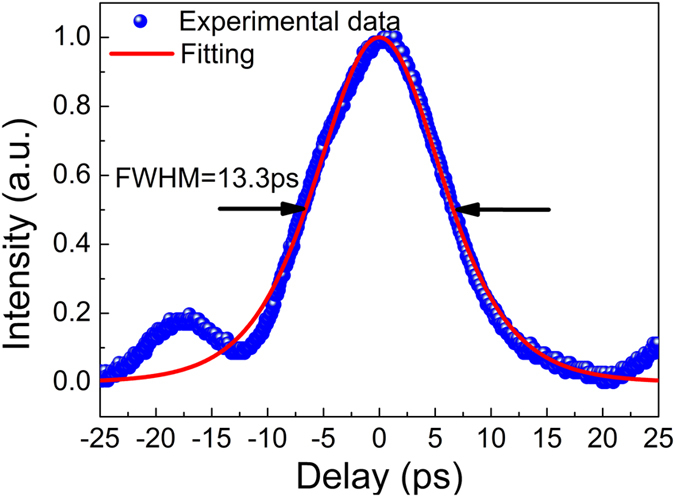
Autocorrelation trace of the mode-locked pulses at the launched pump power of 512.3 mW recorded by the digital oscilloscope. The blue points are the experimental data and the red line is the fitting result.

In the first system, only Q-switching was obtained except the CW laser at the low pump range. To demonstrate the potential of BP in mode-locking around 3 μm, another carefully designed fiber laser was constructed as shown in Fig. 3b. In this case, the CW laser was also firstly generated as the launched pump power was increased to 66.4 mW. The much lower laser threshold than that in the previous case was mainly attributed to the use of high feedback output coupler (OC) and high gain fiber. Unlike the previous case, when the launched pump power reached 190.1 mW, the CW operation state was broken by stable Q-switched mode-locking (QSML) as shown in Fig. 5a instead of Q-switching. The inset of Fig. 5a shows the single Q-switched envelope involving mode-locked components. At this pump level, the repetition rate of 9.43 kHz and duration of 32.7 μs of the Q-switched envelope were achieved. Further increasing the launched pump power, the laser was still operated at the QSML state with the increased repetition rate, decreased duration and weakened amplitude fluctuation of the Q-switched envelope. At the launched pump power of 383.42 mW, the pulse train and single Q-switched envelope were recorded as shown in Fig. 5b and its inset, respectively. The highest repetition rate of 207.6 kHz and shortest duration of 2.74 μs of the Q-switched envelope were achieved. Once the launched pump power was beyond this level, the laser instantly switched to the continuous wave mode-locking (CWML) state as shown in Fig. 5c. The left and right insets inside show the temporal views on the oscilloscope at a scanning span of 700 ns and 70 ns, respectively. The interval of 71.5 ns between adjacent pulses matched well with the cavity round trip time indicating the laser was operated at the single pulse state. Note that the displayed pulse duration of 14.5 ns was not the true level due to the limited operation bandwidth of the used InAs detector. This stable CWML state can be maintained until the launched pump power of 512.3 mW. Once beyond this pump, however, the CWML became very unstable and then quickly degraded into CW operation state when the launched pump power was increased to 1.43 W as a result of pulse breaking at high pump power level. The CW state can be maintained until the maximum available launched pump power of 2.99 W. If the pump was adjusted back to 512.3 mW, the stable CWML reappeared indicating the BP was not damaged. Figure 5d shows the output power as a function of the launched pump power. It is observed that the output power increases almost linearly with the launched pump power at a slope efficiency of 19.5%. At the launched pump power of 512.3 mW, the maximum pulsed output power was measured to be 87.8 mW giving the calculated maximum pulse energy of 6.28 nJ. Moreover, the optical spectrum of the mode-locked pulses was also measured at this pump level as shown in Figure 5e. The center wavelength and FWHM were 2866.7 nm and 4.35 nm, respectively. Note that though our system was operated at anomalous dispersion regime, no typical Kelly sidebands of solitons were observed on the spectrum. It might be attributed to the relatively small self-phase modulation which cannot initiate the soliton mode-locking^69^. To exclude the possibility of self-pulsing, the focused beam spot was moved onto the clean region of the BP partially coated Au mirror as before. The obtained CW operation state indicated that the mode-locking was indeed resulted from the function of the BP. At this moment, the optical spectrum was measured as a comparison as shown in Figure 5e. It is seen that the center wavelength was red-shifted to 2868.9 nm while the FWHM was significantly reduced to 1.15 nm due to less Fourier spectral components required. Figure 5f shows the RF spectrum of the mode-locked pulses at a narrow scanning range of 1 MHz with a resolution bandwidth of 10 kHz. The measured repetition rate of 13.987 MHz matched well with the calculated effective cavity length (7.1 m gain fiber length and extra 0.2 m free-space length). The measured SNR of 56 dB indicated it was the stable mode-locking. The RF spectrum at a wide span from 0 MHz to 500 MHz is also shown in the inset of Fig. 5f. The smooth roll-off of the clean harmonic frequency components suggested no multiple pulse operation existed. Figure 6 shows the autocorrelation trace recorded by the digital oscilloscope yielding the FWHM of 13.3 ps. The observed side wing was resulted from the background noise on the detection system instead of optical signal^24^. Assuming the trace approximated a sech^2^ function, the mode-locked pulse duration was calculated to be 8.6 ps. It gave the time bandwidth product (TBP) of 1.366 indicating the mode-locked pulses were strongly chirped.

## Discussion

In the above experiments, two fiber lasers with different cavity structures and gain fibers were designed to successfully obtain Q-switching and mode-locking, respectively using the same BP as the SA. Besides, another two relative experiments were also performed to illustrate the significant influence of intra-cavity pulse energy on the laser operation state. In the first experiment above, if the DM that has a reflection of 20% at 2.9 μm was introduced into the cavity as the OC while keeping the other arrangements unchanged, only Q-switching was gained just with a decreased slope efficiency as a result of less laser output. It indicated that the 20% feedback was not strong enough to compensate the low gain resulting from the Ho^3+^-doped fluoride fiber, therefore the intra-cavity pulse energy still cannot reach the level capable of yielding mode-locking. In the second experiment above, once the DM as the OC was removed away from the cavity while perpendicularly cleaving the fiber end close to the pump (i.e., 4% Fresnel reflection acting as the feedback), the CWML was absent owing to the decreased intra-cavity pulse energy. These results suggested that intra-cavity pulse energy was indeed closely related to the laser temporal operation state. Furthermore, the long-term stability of BP induced pulse generation is also attractive. It is well known that BP is very hydrophilic and will interact with oxygen and water molecules if exposed to air. In our experiments, the pulsed fiber lasers operating in either Q-switching or mode-locking regime could be working stably within about 3 days which were mainly limited by the deterioration of the used BP. In the future, a series of infrared transparent organic materials (e.g., polymethyl methacrylates, polyvinyl acetate, etc.) would be used to mix with BP for improving its long-term stable performance.

### Conclusions

In summary, we have successfully demonstrated the potential of BP in pulse generation in near 3 μm wavelength mid-infrared region. Based on the widely used LPE method, high quality BP material was fabricated and characterized by the way of Raman spectrum measurement, TEM, AFM. Moreover, its saturable absorption property was also investigated at 2 μm using the typical balanced twin-detector measurement method. The modulation depth of 41.2% and saturation peak intensity of 3.767 MW/cm^2^ (or saturation pulse fluence of 15.821 μJ/cm^2^) were achieved. Using BP as the SA, the passively Q-switched singly Ho^3+^-doped fluoride fiber laser emitting at 2970.3 nm was constructed with the aid of 4% Fresnel reflection as the OC feedback. The maximum output power of 308.7 mW at a slope efficiency of 11.35% and single pulse energy of 4.93 μJ were obtained. The achieved repetition rate and pulse duration were 62.5 kHz and 2.41 μs, respectively. While the passively mode-locked Ho^3+^/Pr^3+^ co-doped fluoride fiber laser centered at 2866.7 nm based on the same BP SA was also established using a dichoic mirror having 20% reflection as the OC. It gave the pulse duration of 8.6 ps at the repetition rate of 13.987 MHz. The achieved output power and pulse energy were 87.8 mW and 6.28 nJ, respectively. These results indicated that a same BP could lead to either Q-switching or mode-locking in different cavities while further extended the record wavelength of BP in pulse generation. In the future, more efforts would be deployed to investigate its potential at longer mid-infrared wavelengths.

## Methods

### Fabrication of black phosphorus SA

The layered BP in our experiments was fabricated using the cost-effective LEP method^71^. Firstly, the bulk BP (15 mg) purchased from Smart Element was added into the saturated NaOH/NMP solution (30 mL). Secondly, the mixture was put in a bath sonicator operated at 40 kHz frequency to be processed for 4 hours for exfoliation of the bulk BP. After the sonication step, the solution was centrifuged at 3000 rpm for 10 min to remove the large size BP. Then the supernatant was further centrifuged at 7000 rpm. Finally, the sediment (0.1 mL) of the second centrifugation step was deposited onto the center region of a commercial Au mirror (Thorlabs) and then put in an oven for drying 1 hour at the temperature of 60 °C to achieve the free-space BP SA. Note the following characterizations including TEM, AFM and Raman spectrum measurement are all based on the used sediment.

### Nonlinear absorption measurement of black phosphorus

**Figure 7:**
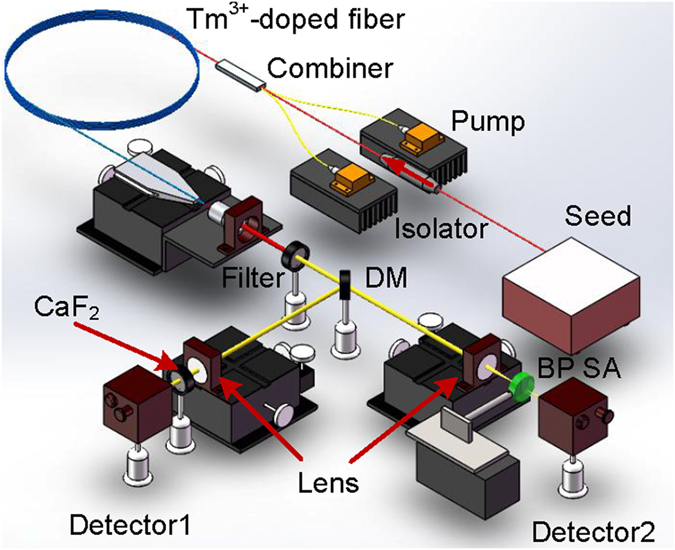
Experimental setup used for BP nonlinear absorption measurement.

A typical Z-scan scheme involving a balance twin-detector arrangement was designed to measure the nonlinear absorption of the BP with reduced measurement errors as shown in Fig. 7. The used source is an in-house 2 μm ultra-short fiber laser including a NPR-based soliton mode-locked Tm^3+^-doped ring oscillator seed and its corresponding MOPA system. Note that selecting the ultra-short laser in this case is to obtain a high peak power with a low pulse energy thus weakening pulsed thermal influence on measurement results. The relative performance parameters are also listed as following: repetition rate of 21.5 MHz, FWHM of ~1 nm, pulse duration of 4.2 ps, center wavelength of 2000.56 nm, signal-to-noise ratio (SNR) of ~60 dB. Its output power can be also adjusted continuously at the range of 0~200 mW by varying the pump power of the amplifier while the amplified soliton mode-locked pulses always maintained at a high SNR of exceeding 50 dB. The output laser was firstly collimated using a standard objective lens and then split into two orthogonal beams by a 45° angle placed DM with respect to the incident beam (splitting ratio is 50:50 at 2 μm). Here a 2 μm bandpass filter was placed between them to remove the residual 793 nm pump. Then both beams were focused by two same plano-convex lenses with a focal length of 40 mm. For the left path, the power of the focused beam after passing through a fixed clean CaF_2_ substrate was monitored by a power meter detector (labeled as Detector 1) to act as the reference. For the right path, the as-prepared BP coated CaF_2_ substrate (note that its fabrication process was almost same as the BP coated Au mirror before including the amount of the used BP) was inserted in the light path while keeping its surface perpendicular to the focused beam axis. Besides the CaF_2_ substrate can be also translated along the beam axial direction by adjusting its connected linear motorized stage. Therefore the size of the laser beam spot on the BP can be varied continuously. Then another power meter detector (labeled as Detector 2) was used to record the power of the laser passing through the BP coated CaF_2_ substrate. In this experiment, the position corresponding to the minimum focused beam spot on the BP was firstly found by the means of monitoring power. Gradually increasing the laser output power, a series of powers extracted by both detectors were recorded. Upon dividing the Detector 2 recorded powers by the reference power (i.e., the Detector 1 recorded power), the nonlinear transmission curve can be achieved.

### Design of mid-infrared pulsed fiber lasers

In our experiments, two laser systems with different gain fibers and cavities were designed to demonstrate the Q-switching and mode-locking, respectively based on the same BP SA. It is well known that, to achieve CWML without Q-switching instability, the following condition should be fulfilled^72^:


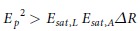


where *E*_*p*_ is the intra-cavity pulse energy, *E*_*sat,L*_ and *E*_*sat,A*_ are the saturation energies of the used fiber and SA, *ΔR* is the modulation depth of the SA. It is observed that if CWML is required, larger intra-cavity pulse energy, smaller saturation energies for the used fiber and SA, and lower modulation depth for the SA are more preferred. On contrary, if this condition is broken, Q-switching is easier to be obtained. According to these criteria, the following fiber lasers were constructed.

Figure 3a shows the schematic diagram of the passively Q-switched singly Ho^3+^-doped fluoride fiber laser using BP as the SA. Two commercially available high power 1150 nm diode lasers (Eagleyard Photonics, Berlin) were exploited to pump the gain fiber after polarization multiplexing via a polarized beam splitter (PBS) and focusing by a 1150 nm AR-coated ZnSe objective lens (Innovation Photonics, LFO-5-6-3 μm, 0.25 NA) with a 6.0 mm focal length acting as the collimator for the light out-coupled from the fiber core as well. A DM (96%T@1150 nm, 95%R@~3 μm) was placed between the PBS and ZnSe objective lens at an angle of 45° with respect to the pump beam to direct the laser output. A 3 μm bandpass filter with a FWHM of 500 nm was utilized to block the residual pump. The gain fiber (Fiberlabs, Japan) was a piece of commercial double-cladding singly Ho^3+^-doped fluoride fiber having a circular-shaped pump core with a diameter of 123 μm and NA of 0.5. The diameter and NA of the fiber core were 10 μm and 0.16, respectively. The dopant concentration was 2.0 mol.%, therefore the selected 5.2 m fiber could provide 92% pump absorption efficiency. In this case, the fiber has relatively large saturation energy as a result of its small emission cross-section at the free-running laser wavelength. Moreover, the relative low gain and slope efficiency caused by the low dopant concentration leads to the low intra-cavity pulse energy. The fiber end close to the pump was perpendicularly cleaved acting as the OC with 4% Fresnel reflection which was also for reducing the cavity feedback hence decreasing the intra-cavity pulse energy. Therefore, the above condition is easy to be broken. While the other end of the fiber was cleaved at an angle of 8° to avoid the parasitic lasing. The laser outputted from the angle cleaved fiber end was collimated using another specifically coating designed ZnSe objective lens with the focal length of 6 mm having a >95% transmission at 3 μm and <10% transmission at 1150 nm. Afterwards, via a commercial ZnSe objective lens (Innovation Photonics, LFO-5-12-3 μm, 0.13 NA) with the focal length of 12 mm, the collimated laser was focused onto the BP partially coated Au mirror (Thorlabs) which acted as both terminated feedback and SA. A high-precision three-dimension adjuster was used to mount the Au mirror to optimize its intra-cavity position.

In order to achieve CWML, the feedback and gain should be increased for larger intra-cavity pulse energy. The experimental setup of mode-locked laser is shown in Fig. 3b. The commercial double-cladding Ho^3+^/Pr^3+^ co-doped fluoride fiber (Fiberlabs, Japan) was selected in this system. It has an octangular pump core with a diameter of 125 μm and NA of 0.5 and a circular core with a diameter of 10 μm and NA of 0.2. The concentration of the Ho^3+^ and Pr^3+^ were 30,000 and 2500 ppm, respectively. The selected fiber length of 7.1 m provided >90% pump absorption efficiency. Note that the high gain of this fiber could give the high intra-cavity pulse energy at the same pump level which was helpful for the generation of CWML. Besides, another DM having a reflection of 20% at 2.9 μm was introduced acting as the OC. It improved the intra-cavity laser intensity by increasing the cavity feedback hence giving an increased tendency to gain CWML. Generally, higher cavity feedback induced higher intra-cavity pulse energy is helpful for achieving CWML, but will lead to the decay of the slope efficiency. For our system, 20% feedback can realize CWML, and thus higher cavity feedback at a sacrifice of the slope efficiency is not necessary. While the fiber end close to the pump was also cleaved at an angle of 8° to avoid the parasitic oscillation.

**Figure 8:**
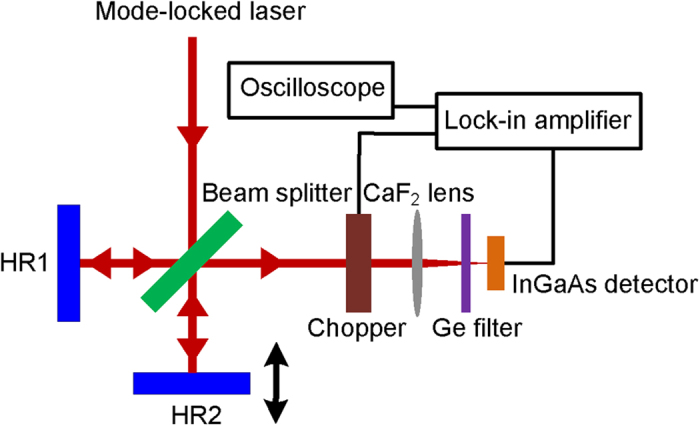
Experimental setup of in-house intensity autocorrelator based on two-photon absorption in InGaAs detector.

In the above experiments, the pulse trains were recorded using an InAs detector with a response time of ~2 ns connected to a 500 MHz digital oscilloscope. A monochromator with a resolution of 0.1 nm (Princeton instrument Acton SP2300) was used to measure the optical spectra of the laser output. A RF spectrum analyzer (Advantest R3267) with an adjustable resolution from 10 Hz to 100 MHz was connected to the InAs detector to measure the RF spectrum hence achieving pulsed repetition rate and signal-to-noise ratio (SNR). The duration of the mode-locked pulse was measured using an in-house intensity autocorrelator as shown in Fig. 8. The incident beam was firstly split into two beams by a 50:50 beam splitter and then directed onto the high-reflectivity (HR) mirrors 1 and 2, respectively. The reflected beams from the HR mirrors intersected again at the beam splitter and combined into an interfered beam which was then focused onto an InGaAs detector by a CaF_2_ lens. Here, the two-photon absorption process in the InGaAs detector was used to generate and detect the autocorrelation signal. In this case, the HR mirror 1 was fixed while HR mirror 2 was mounted on a motorized translation stage to vary the temporal overlap of the reflected pulses from two HR mirrors. To prevent residual pump and room light from interfering with the measurement, a Ge filter was placed in front of the InGaAs detector. An optical beam chopper and lock-in amplifier were used to accurately measure the weak two-photon signal generated from the InGaAs detector. A digital oscilloscope was connected to the lock-in amplifier to record autocorrelation trace.

## Additional Information

**How to cite this article**: Li, J. *et al.* Black phosphorus: a two-dimension saturable absorption material for mid-infrared Q-switched and mode-locked fiber lasers. *Sci. Rep.* **6**, 30361; doi: 10.1038/srep30361 (2016).

## References

[b1] Pile D., Horiuchi N., Won RPCh. & Graydon O. Extending opportunities. Nat. Photon. 6, 407 (2012).

[b2] Titte F. K., Wysocki G., Kosterev A. & Bakhirkin Y. [Semiconductor Laser Based Trace Gas Sensor Technology: Recent Advances and Applications] Mid-Infrared Coherent Sources and Applications [Ebrahim-Zadeh M. & Sorokina I. T. (ed)] [467–493] (Springer, Berlin, 2008).

[b3] Waechter H. & Sigrist M. [Trace Gas Analysis with Isotopic Selectivity Using DFG-Sources] Mid-Infrared Coherent Sources and Applications [Ebrahim-Zadeh M. & Sorokina I. T. (ed)] [495–510] (Springer, Berlin, 2008).

[b4] Ngai A. K. Y., Persijn S. T., van Herpen M. M. J. W., Cristescu S. M. & Harren F. J. M. [Photoacoustic Spectroscopy Using Continuous Wave Optical Parametric Oscillators] Mid-Infrared Coherent Sources and Applications [Ebrahim-Zadeh M. & Sorokina I. T. (ed)] [511–533] (Springer, Berlin, 2008).

[b5] Mürtz M. & Hering P. [Online Monitoring of Exhaled Breath Using Mid-Infrared Laser Spectroscopy] Mid-Infrared Coherent Sources and Applications [Ebrahim-Zadeh M. & Sorokina I. T. (ed)] [535–555] (Springer, Berlin, 2008).

[b6] Sorokin E. [Ultrabroadband Solid-State Lasers in Trace Gas Sensing] Mid-Infrared Coherent Sources and Applications [Ebrahim-Zadeh M. & Sorokina I. T. (ed)] [557–574] (Springer, Berlin, 2008).

[b7] Steiner R. [Medical Applications of Mid-IR Solid-State Lasers] Mid-Infrared Coherent Sources and Applications [Ebrahim-Zadeh M. & Sorokina I. T. (ed)] [575–588] (Springer, Berlin, 2008).

[b8] Ivanov M., Yakovlev Vlad. & Krausz F. [Opportunities for Mid-IR Sources in Intense-Field and Attosecond Physics] Mid-Infrared Coherent Sources and Applications [Ebrahim-Zadeh M. & Sorokina I. T. (ed)] [589–598] (Springer, Berlin, 2008).

[b9] Sorokina I. T. [Crystalline Mid-Infrared Lasers] Solid-State Mid-Infrared Laser Sources [Sorokina I. T. & Vodopyanov K. L. (ed)] [262–358] (Springer, Berlin, 2003).

[b10] Yao Y., Hoffman A. J. & Gmachl C. F. Mid-infrared quantum cascade lasers. Nat. Photon. 6, 432–439 (2012).

[b11] Cornelia F. & Markus W. S. [Mid-IR Difference Frequency Generation] Solid-State Mid-Infrared Laser Sources [Sorokina I. T. & Vodopyanov K. L. (ed)] [99–143] (Springer, Berlin, 2003).

[b12] Konstantin V. [Pulsed Mid-IR Optical Parametric Oscillators] Solid-State Mid-Infrared Laser Sources [Sorokina I. T. & Vodopyanov K. L. (ed)] [144–224] (Springer, Berlin, 2003).

[b13] Jackson S. D. Towards high-power mid-infrared emission from a fibre laser. Nat. Photon. 6, 423–431 (2012).

[b14] Fortin V., Bernier M., Bah S. T. & Vallée R. 30 W fluoride glass all-fiber laser at 2.94 μm. Opt. Lett. 40, 2882–2885 (2015). 26076286 10.1364/OL.40.002882

[b15] Bernier M., Fortin V., El-Amraoui M., Messaddeq Y. & Vallée R. 3.77 μm fiber laser based on cascaded Raman gain in a chalcogenide glass fiber. Opt. Lett. 39, 2052–2055 (2014). 24686672 10.1364/OL.39.002052

[b16] Yao C. F. *et al.* Holmium-doped fluorotellurite microstructured fibers for 2.1 μm lasing. Opt. Lett. 40, 4695–4698 (2015). 26469597 10.1364/OL.40.004695

[b17] Carbonnier C., Többen H. & Unrau U. B. Room temperature CW fibre laser at 3.22 μm. Electron. Lett. 34, 893–894 (1998).

[b18] Tobben H. Room temperature cw fibre laser at 3.5 μm in Er^3+^-doped ZBLAN glass. Electron., Lett. 28, 1361–1363 (1992).

[b19] Henderson-Sapir O., Munch J. & Ottaway D. J. Mid-infrared fiber lasers at and beyond 3.5 μm using dual-wavelength pumping. Opt. Lett. 39, 493–496 (2014). 24487848 10.1364/OL.39.000493

[b20] Schneider J., Carbonnier C. & Unrau U. B. Characterization of a Ho^3+^-doped fluoride fiber laser with a 3.9 μm emission wavelength. Appl. Opt. 36, 8595–8600 (1997). 18264407 10.1364/ao.36.008595

[b21] Henderson-Sapir O., Jackson S. D. & Ottaway D. J. Versatile and widely tunable mid-infrared erbium. Opt. Lett. 41, 1676–1679 (2016). 27192316 10.1364/OL.41.001676

[b22] Fortin V., Maes F., Bernier M., Bah S. T., D’Auteuil M. & Vallée R. Watt-level erbium-doped all-fiber laser at 3.44 μm. Opt. Lett. 41, 559–562 (2016). 26907423 10.1364/OL.41.000559

[b23] Li J. F. *et al.* Semiconductor saturable absorber mirror passively Q-switched 2.97 μm fluoride fiber laser. Laser Phys. Lett. 11, 065102 (2014).

[b24] Li J. F., Hudson D. D., Liu Y. & Jackson S. D. Efficient 2.87 μm fiber laser passively switched using a semiconductor saturable absorber mirror. Opt. Lett. 37, 3747–3749 (2012). 23041846 10.1364/ol.37.003747

[b25] Tang P. H. *et al.* Watt-level passively mode-locked Er^3+^-doped ZBLAN fiber laser at 2.8 μm. Opt. Lett. 40, 4855–4858 (2015). 26512467 10.1364/OL.40.004855

[b26] Li J. F. *et al.* Mid-infrared passively switched pulsed dual wavelength Ho^3+^-doped fluoride fiber laser at 3 μm and 2 μm. Sci. Rep. 5, 10770 (2015). 26041105 10.1038/srep10770 PMC4455194

[b27] Haboucha A., Fortin V., Bernier M., Genest J., Messaddeq Y. & Vallée R. Fiber Bragg grating stabilization of a passively mode locked 2.8 μm Er^3+^: fluoride glass fiber laser. Opt. Lett. 39, 3294–3297 (2014). 24876036 10.1364/OL.39.003294

[b28] Hu T., Hudson D. D. & Jackson S. D. Stable, self-starting, passively mode-locked fiber ring laser of the 3 μm class. Opt. Lett. 39, 2133–2136 (2014). 24686693 10.1364/OL.39.002133

[b29] Wei C., Zhu X. S., Norwood R. A. & Peyghambarian N. Passively Q-Switched 2.8-μm nanosecond fiber laser. IEEE Photon. Technol. Lett. 24, 1741–1744 (2012).

[b30] Li J. F., Luo H. Y., Wang L. L., Zhai B., Li H. P. & Liu Y. Tunable Fe^2+^:ZnSe passively Q-switched Ho^3+^-doped ZBLAN fiber laser around 3 μm. Opt. Expess 23, 22362–22370 (2015). 10.1364/OE.23.022362 26368206

[b31] Wei C., Zhu X. S., Norwood R. A. & Peyghambarian N. Passively continuous-wave mode-locked Er^3+^-doped ZBLAN fiber laser at 2.8 μm. Opt. Lett. 37, 3849–3851 (2012). 23041880 10.1364/OL.37.003849

[b32] Duval S., Bernier M., Fortin V., Genest J., Piché M. & Vallée R. Femtosecond fiber lasers reach the mid-infrared. Optica 2, 623–626 (2015).

[b33] Hu T., Jackson S. D. & Hudson D. Ultrafast pulses from a mid-infrared fiber laser. Opt. Lett. 40, 4226–4228 (2015). 26371902 10.1364/OL.40.004226

[b34] Xia F. N., Wang H., Xiao D., Dubey M. & Ramasubramaniam A. Two-dimensional material nanophotonics. Nat. Photon. 8, 899–907 (2014).

[b35] Bao Q. L. *et al.* Atomic-layer graphene as a saturable absorber for ultrafast pulsed lasers. Adv. Funct. Mater. 19, 3077–3083 (2009).

[b36] Hasan T. *et al.* Nanotube-polymer composites for ultrafast photonics. Adv. Mater. 21, 3874–3899 (2009).

[b37] Sun Z. *et al.* A stable, wideband tunable, near transform-limited, graphene-mode-locked, ultrafast laser. Nano. Res. 3, 653–660 (2010). 10.1021/nn901703e 20099874

[b38] Liu J., Wu S. D., Yang Q. H. & Wang P. Stable nanosecond pulse generation from a graphene-based passively Q-switched Yb-doped fiber laser. Opt. Lett. 36, 4008–4010 (2011). 22002368 10.1364/OL.36.004008

[b39] Zhao L., Tang D. Y., Zhang H., Wu X., Bao Q. L. & Loh K. P. Dissipative soliton operation of an ytterbium-doped fiber laser mode locked with atomic multilayer graphene. Opt. Lett. 35, 3622–3624 (2010). 21042370 10.1364/OL.35.003622

[b40] Liu J., Xu J. & Wang P. Graphene-based passively Q-switched 2 μm thulium-doped fiber laser. Opt. Commun. 285, 5319–5322 (2012).

[b41] Sobon G., Sotor J., Pasternak I., Krajewska A., Strupinski W. & Abramski K. M. Thulium-doped all-fiber laser mode-locked by CVD graphene/PMMA saturable absorber. Opt. Express 21, 127971–127976 (2013). 10.1364/OE.21.012797 23736498

[b42] Zhu G. W. *et al.* Graphene mode-locked fiber laser at 2.8 μm. IEEE Photon. Technol. Lett. 28, 7–10 (2016).

[b43] Wei C. *et al.* Graphene Q-switched 2.78 μm Er^3+^-doped fluoride fiber laser. Opt. Lett. 38, 3233–3236 (2013). 23988922 10.1364/OL.38.003233

[b44] Nair R. R. *et al.* Fine structure constant defines visual transparency of graphene. Science 320, 1308 (2008). 18388259 10.1126/science.1156965

[b45] Zhang H. *et al.* Molybdenum disulfide (MoS_2_) as a broadband saturable absorber for ultra-fast photonics. Opt. Express 22, 7249–7260 (2014). 24664073 10.1364/OE.22.007249

[b46] Liu H. *et al.* Femtosecond pulse erbium-doped fiber laser by a few-layer MoS_2_ saturable absorber. Opt. Lett. 39, 4591–4594 (2014). 25078236 10.1364/OL.39.004591

[b47] Du J. *et al.* Ytterbium-doped fiber laser passively mode locked by few-layer Molybdenum Disulfide (MoS_2_) saturable absorber functioned with evanescent field interaction. Sci. Rep. 4, 6346 (2014). 25213108 10.1038/srep06346 PMC4161963

[b48] Mao D. *et al.* WS_2_ mode-locked ultrafast fiber laser. Sci. Rep. 5, 7965 (2015). 25608729 10.1038/srep07965 PMC4302320

[b49] Yan P. G. *et al.* Passively mode-locked fiber laser by a cell-type WS_2_ nanosheets saturable absorber. Sci. Rep. 5, 12587 (2015). 26213180 10.1038/srep12587 PMC4515829

[b50] Lin J., Hu Y. Y., Chen C. J., Gu C. & Xu L. X. Wavelength-tunable Yb-doped passively Q-switching fiber laser based on WS_2_ saturable absorber. Opt. Express 23, 29059–29064 (2015). 26561175 10.1364/OE.23.029059

[b51] Chen B. H., Zhang X. Y., Wu K., Wang H., Wang J. & Chen J. P. Q-switched fiber laser based on transition metal dichalcogenides MoS_2_, MoSe_2_, WS_2_, and WSe_2_. Opt. Express 23, 26723–26737 (2015). 26480185 10.1364/OE.23.026723

[b52] Luo Z. Q. *et al.* 1-, 1.5-, and 2-μm fiber lasers Q-switched by a broadband few-layer MoS_2_ saturable absorber. IEEE J. Lightwave Technol. 32, 4077–4084 (2014).

[b53] Jung M. W., Lee J. S., Park J., Koo J., Jhon Y. M. & Lee J. H. Mode-locked, 1.94-μm, all-fiberized laser using WS_2_-based evanescent field interaction. Opt. Express 23, 19996–20006 (2015). 26367658 10.1364/OE.23.019996

[b54] Hasan M. Z. & Kane C. L. Colloquium: Topological insulators. Rev. Mod. Phys. 82, 3045–3067 (2010).

[b55] Zhao C. J. *et al.* Ultra-short pulse generation by a topological insulator based saturable absorber. Appl. Phys. Lett. 101, 21106 (2012).

[b56] Chen Y. *et al.* Self-assembled topological insulator: Bi_2_Se_3_ membrane as a passive Q-switcher in an erbium-doped fiber laser. J. Lightwave Technol. 31, 2857–2863 (2013).

[b57] Li J. F. *et al.* 3-μm mid-infrared pulse generation using topological insulator as the saturable absorber. Opt. Lett. 40, 3659–3662 (2015). 26258382 10.1364/OL.40.003659

[b58] Xia F., Wang H. & Jia Y. Rediscovering black phosphorus as an anisotropic layered material for optoelectronics and electronics. Nat. Commun. 5, 4458 (2014). 25041752 10.1038/ncomms5458

[b59] Yang W. Q. *et al.* Ultrafast recovery time and broadband saturable absorption properties of black phosphorus suspension. Appl. Phys. Lett. 107, 091905–1–5 (2015).

[b60] Lin S. H. *et al.* Solution-processable ultrathin black phosphorus as an effective electron transport layer in organic photovoltaics. Adv. Funct. Mater. 26, 864–871 (2016).

[b61] Liu H., Du Y. C., Deng Y. X. & Ye P. D. Semiconducting black phosphorus: Synthesis, transport properties and electronic applications. Chem. Soc. Rev. 44, 2732–2743 (2015). 25307017 10.1039/c4cs00257a

[b62] Tran V., Soklaski R., Liang Y. & Yang L. Layer-controlled band gap and anisotropic excitons in few-layer black phosphorus. Phys. Rev. B 89, 235319 (2014).

[b63] Lu S. B. *et al.* Broadband nonlinear optical response in multilayer black phosphorus: an emerging infrared and mid-infrared optical material. Opt. Express 23, 11183–11194 (2015). 25969214 10.1364/OE.23.011183

[b64] Chen Y. *et al.* Mechanically exfoliated black phosphorus as a new saturable absorber for both Q-switching and mode-locking laser operation. Opt. Express 23, 12823–12833 (2015). 26074536 10.1364/OE.23.012823

[b65] Mu H. R. *et al.* Pulsed lasers: Black phosphorus-polymer composites for pulsed lasers. Adv. Opt. Mater. 3, 1446–1446 (2015).

[b66] Chen Y., Mu H. R., Li P. F., Lin S. H., Shivananju B. N. & Bao Q. L. Optically driven black phosphorus as a saturable absorber for mode-locked laser pulse generation. Opt. Eng. 55, 081317-1-6 (2016).

[b67] Sotor J., Sobon G., Kowalczyk M., Macherzynski W., Paletko P. & Abramski K. M. Ultrafast thulium-doped fiber laser mode locked with black phosphorus. Opt. Lett. 40, 3885–3888 (2015). 26274685 10.1364/OL.40.003885

[b68] Qin Z. P. *et al.* Black phosphorus as saturable absorber for the Q-switched Er:ZBLAN fiber laser at 2.8 μm. Opt. Express 23, 24713–24718 (2015). 26406672 10.1364/OE.23.024713

[b69] Qin Z. P., Xie G. Q., Zhao C. J., Wen S. C., Yuan P. & Qian L. J. Mid-infrared mode-locked pulse generation with multilayer black phosphorus as saturable absorber. Opt. Lett. 41, 56–58 (2016). 26696157 10.1364/OL.41.000056

[b70] Tran V., Soklaski R., Liang Y. & Yang L. Layer-controlled band gap and anisotropic excitons in few-layer black phosphorus. Phys. Rev. B 89, 235319 (2014).

[b71] Guo Z. N. *et al.* From black phosphorus to phosphorene: basic solvent exfoliation, evolution of Raman scattering, and applications to ultrafast photonics. Adv. Funct. Mater. 25, 6996–7002 (2015).

[b72] Hönninger C., Paschotta R., Morier-Genoud F., Moser M. & Keller U. Q-switching stability limites of continuous-wave passive mode locking. J. Opt. Soc. Am. B 16, 46–56 (1999).

